# Heat Shock Proteins in Association with Heat Tolerance in Grasses

**DOI:** 10.1155/2011/529648

**Published:** 2011-02-24

**Authors:** Yan Xu, Chenyang Zhan, Bingru Huang

**Affiliations:** ^1^Department of Plant Biology and Pathology, Rutgers University, New Brunswick, NJ 08901, USA; ^2^Department of Biology, Ramapo College of New Jersey, NJ 07430, USA; ^3^Department of Biochemistry, Albert Einstein College of Medicine, Bronx, NY 10461, USA

## Abstract

The grass family Poaceae includes annual species cultivated as major grain crops and perennial species cultivated as forage or turf grasses. Heat stress is a primary factor limiting growth and productivity of cool-season grass species and is becoming a more significant problem in the context of global warming. Plants have developed various mechanisms in heat-stress adaptation, including changes in protein metabolism such as the induction of heat shock proteins (HSPs). This paper summarizes the structure and function of major HSPs, recent research progress on the association of HSPs with grass tolerance to heat stress, and incorporation of HSPs in heat-tolerant grass breeding.

## 1. Introduction

The grass family Poaceae is comprised of nearly 10,000 species, including annual species cultivated as major grain crops and perennial species cultivated as forage for livestock or turf grasses on home lawns, commercial landscapes, roadsides, parks, athletic fields, and golf courses. Based on the ranges of temperature and precipitation that grasses adapt to, they are classified into warm-season and cool-season categories. Cool-season and warm-season grasses have distinctive photosynthetic pathways, referred as C_3_ and C_4_ pathways, respectively. C_3_ or cool-season grass species grow most actively at temperatures ranging from 18°C–24°C while C_4_ or warm-season grass species have an optimum growth temperature between 30°C –35°C [1]. Heat stress is particularly detrimental to cool-season grass species. 

Temperature rises beyond a threshold (5°C –10°C above ambient) may cause irreversible damages to plant function and development or alteration of metabolism, resulting in reduction in growth and yield production [2]. The extent to which heat stress causes damage on plants is a complex issue. It depends on the intensity, duration and rate of increase in temperature, as well as other environmental conditions, such as when the high temperature occurs (during the day or the night) and where it occurs (in the air or the soil) [3, 4]. Since predicted global warming has become a serious threat for sustainable agriculture worldwide, an increasing challenge has been imposed to improve grass tolerance to high temperature.

## 2. Mechanisms for Heat Tolerance in Grasses

Grass may survive heat stress through heat-avoidance or heat-tolerance mechanisms [5]. Heat avoidance is the ability of plants to maintain internal temperatures below lethal stress levels, including transpirational cooling, changes in leaf orientation, reflection of solar radiation, leaf shading of tissues that are sensitive to sunburn, and extensive rooting [6, 7]. However, heat-avoiding cultivars thriving in the low humidity may lack heat resistance in humid areas due to reduced cooling effects of transpiration [8]. Heat tolerance is the ability of plants to survive high internal tissue temperatures. Heat-tolerant cultivars can be resistant in both humid and arid conditions. Plant tolerance to high temperature may be achieved through various mechanisms, including changes at the molecular, cellular, biochemical, physiological, and whole-plant levels [3, 9]. Typically, heat-tolerant grass species and cultivars exhibit higher activity in the photosynthetic apparatus [10–13] and higher carbon allocation and nitrogen uptake rates [14, 15] when exposed to supraoptimal temperature. Heat stress was found to induce oxidative stress in grasses so that species and cultivars variations in the activities of antioxidant enzymes were associated with differences in heat tolerance [16–19]. Major hormones such as cytokinins and ethylene are also found to play regulatory roles in heat tolerance of grasses [20–23]. 

Heat stress has significant effects on protein metabolism, including degradation of proteins, inhibition of protein accumulation, and induction of certain protein synthesis, depending on the level and duration of heat stress [24, 25]. Moderate heat response involves downregulation of proteins functioning in lipid biogenesis, cytoskeleton structure, sulfate assimilation, amino acid biosynthesis, nuclear transport and antioxidant response [26, 27]. While synthesis of most normal proteins and mRNAs is inhibited in heat stress conditions, the transcription and translation of a small set of proteins, called heat shock proteins (HSPs), may be induced or enhanced when plants are exposed to elevated temperatures [28–30]. 

This paper summarizes the structure and function of major HSPs, recent research progress on the association of HSPs with heat tolerance in grasses, and how knowledge of HSPs may facilitate heat-tolerant grass breeding.

## 3. Structure and Function of Major HSPs

HSPs are generally classified into five evolutionarily conserved groups: HSP100, HSP90, HSP70, HSP60, and small HSPs (sHSPs) [31]. Most, but not all, heat shock proteins are molecular chaperones, which bind and stabilize proteins at intermediate stages of folding, assembly, degradation, and translocation across membranes. The following paragraphs provide more details on the structure and function of each HSP group. 

### 3.1. HSP70

HSP70 proteins compose a large family of highly conserved molecular chaperones widely found in almost all organisms [32]. The sequence identity between bacterial and eukaryotic HSP70s is about 50%, suggesting its critical functions in various life forms [32, 33]. Most eukaryotic organisms have multiple HSP70 homologs, located in diverse cell compartments including cytosol, mitochondrion, chloroplast and endoplasmic reticulum [33]. In addition to their known function in preventing protein aggregation and assisting refolding of nonnative proteins in unfavorable environments, many HSP70 proteins also play essential roles in housekeeping activities under normal conditions. As a good example, in addition to the stress-inducible HSP70s, some HSP70 homologs, which are so called heat shock cognate 70 (HSC70), are constitutively expressed in the eukaryotic cytosol. HSC70 stabilizes nascent proteins being released from ribosomes, preventing possible misfolding and aggregation of partially synthesized polypeptide chains before the end of protein expression [33–35]. A comprehensive expression profile analysis of the Arabidopsis (Arabidopsis thaliana) HSP70 gene family detected 2-20-fold induction of eleven HSP70 genes while the expression of another two HSP70 genes was not enhanced by heat shock treatment [36].

**Figure 1:**
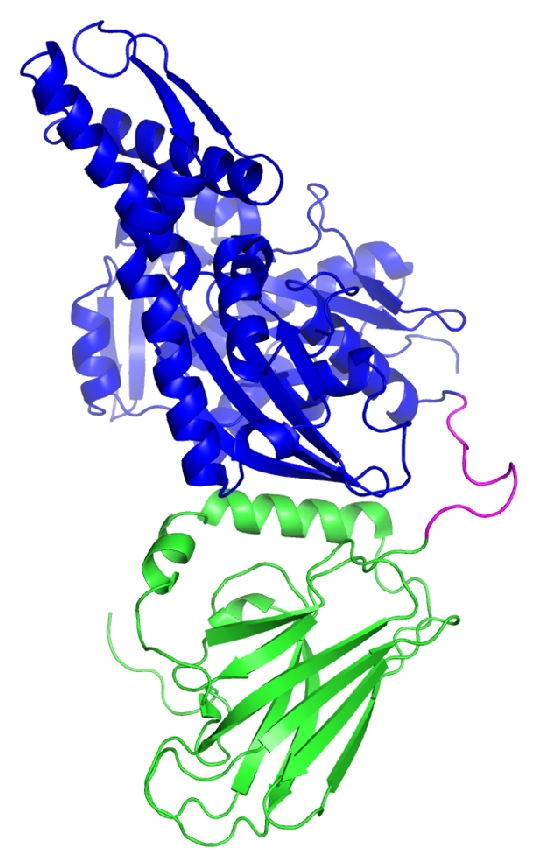
Crystal structure of bovine HSC70. The crystal structure of bovine HSC70 reported by Jiang et al. is shown as ribbon representations (PDB ID: 1YUW) [37]. The N-terminal ATPase domain, interdomain linker, and C-terminal substrate-binding domain are colored in blue, magenta, and green, respectively.

Despite the versatile functions, all HSP70 proteins in higher eukaryotes including plants share similar structures. As represented by a bovine HSC70, the typical structure of HSP70 homolog proteins is composed of an N-terminal ATPase domain about 45 kDa (Blue, Figure 1) and a C-terminal substrate-binding domain about 25 kDa (Green, Figure 1), which are joined by an interdomain linker (Magenta, Figure 1) [37]. The ATPase domain resembles the structure of actin [38] and shares about 64% sequence identity among all eukaryotic HSP70s [34]. The substrate-binding domain has relatively low-sequence conservation (~43% identity) but generally binds short stretches of hydrophobic peptides, which are normally buried inside the folded proteins [34]. The affinity for substrate peptide binding is mediated by different nucleotide-binding states of the ATPase domain. ATP-bound HSP70 binds and releases substrates at fast rates. As the ATP is hydrolyzed to ADP, an allosteric conformational change occurs between the two domains of HSP70, resulting in a higher substrate binding affinity [37, 39, 40]. The switch of HSP70 nucleotide states is facilitated by J-domain cochaperones (HSP40) [35]. This process enables HSP70 to go through cycles of substrate binding and releasing in an ATP-dependent manner, which stabilizes the exposed hydrophobic segments of nonnative proteins, prevents aggregation, and assists the correct folding [34, 41].

### 3.2. HSP60

HSP60, known as chaperonin 60 (cpn60), is one of the first molecular chaperones identified [28]. Similar to HSP70, HSP60 also facilitates ATP-dependent protein folding. Although the two HSP families share partially overlapping functions [34], their structures and mechanisms are distinct.

**Figure 2:**
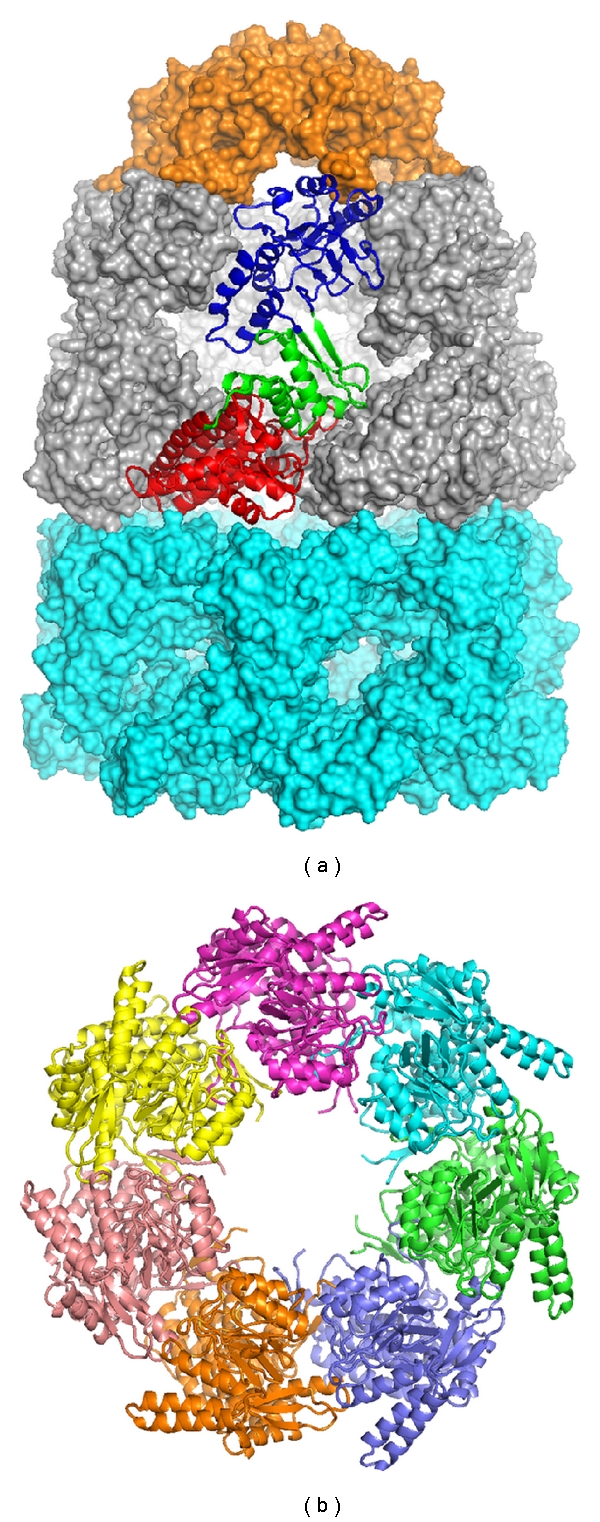
Crystal structure of *E. coli* GroEL and GroES complex. (a) The overall structure of GroEL/GroES complex reported by Xu et al. (PDB ID: 1AON) [42]. The GroES molecule is represented by orange surface. One GroEL monomer in the GroEL top (*cis*) ring is displayed as ribbon, with the apical, intermediate, and equatorial domains colored in blue, green, and red, respectively. The rest of the top (*cis*) ring and the entire bottom (*trans*) ring are shown as grey and cyan surface representations, respectively. (b) Ribbon representation of the GroEL heptameric bottom (*trans*) ring. Each GroEL monomers is shown in a different color.

The best-characterized HSP60 protein is GroEL from *E. coli*. GroEL-like HSP60 homologs have been found in mitochondrion and chloroplast of plant cells but not in cytosol [35]. Under heat-shock conditions, expression of mitochondrion HSP60 is induced and protects preexisting proteins in the organelle from denaturation or inactivation [46], whereas expression of chloroplast HSP60 (chHSP60) is constitutively produced with only modest increase [47]. Due to the high sequence similarity between plant HSP60 and GroEL, the structure of GroEL reported by Xu et al. [42] is shown in Figure 2 to represent the typical assembly of HSP60. GroEL forms a huge homo-oligomer that is composed of two stacked rings, with each ring containing 7 monomers. Each GroEL monomer is about 58 kDa and can be divided into three separate domains: a nucleotide-binding equatorial domain (red in Figure 2(a)), a flexible apical domain (blue in Figure 2(a)), and a hinge-like intermediate domain (green in Figure 2(a)) [48]. The unique structure of GroEL-like HSP60 homo-oligomeric complex creates a hydrophobic cavity about 50 Å in the center of each stacked ring (Figure 2(b)), allowing the accommodation of unfolded polypeptides with size ranging from 10 to 60 kDa [34, 49]. Following the entry of substrates (nonnative proteins), GroEL binds ATP and associates with a 10 kDa cochaperone and GroES (chaperonin 10) (orange in Figure 2(a)) [42]. GroES forms a heptametric ring and interacts with the apical domain of GroEL, acting like a dome-shaped lid that closes the central cavity in the chaperonin. The GroEL/GroES association traps nonnative proteins in an enclosed hydrophobic environment that is amenable to proper folding. Subsequently, the hydrolysis of ATP leads to GroEL conformational change and dissociation of GroES. This results in the release of encapsulated substrates and initiates the next cycle of substrate binding and folding [34]. A primary mechanistic distinction between HSP60 and HSP70 is that HSP60 is capable of binding an entire domain or complete protein, unlike HSP70 that recognizes only short peptide segments.

It is worth noting that chHSP60 is also named as “Rubisco large subunit binding protein” as it plays an essential role in the folding and assembly of Rubisco large subunits [50]. Despite the overall sequence and structural similarity (40%~50% sequence identity) to GroEL, chHSP60 is a hetero-olgiomeric protein complex consisting of *α* and *β* subunits [51]. It has been found that functional Rubisco protein can only be expressed in *E. coli *in the presence of GroEL/GroES system [33]. This suggests that chHSP60 probably facilitates Rubisco folding in a similar manner as its probacterial homolog. 

### 3.3. HSP90 and HSP100

In accordance to HSP70 and HSP60 families, HSP90 proteins are also ATP-dependent molecule chaperones widely expressed in most organisms [52]. However, HSP90 features unique substrate specificity. Instead of binding a wide spectrum of unfolded proteins, HSP90 only interacts with relatively well-folded proteins involved in transcription regulation and signal transduction pathways [53, 54]. Furthermore, the function of HSP90 requires the formation of large protein complexes involving multiple cochaperones, including HSP70 and HSP40, which indicates close cooperation between different molecule chaperone families [54]. Although structural information about plant HSP90 is sparse, high-sequence conservations of the protein family across the phylogeny suggests a similar functional mechanism. Typically, a HSP90 protein is composed of an N-terminal ATPase domain, a middle substrate-protein-binding domain, and a C-terminal dimerization domain [54]. It is proposed that the ATP binding and hydrolysis regulate different conformational states of HSP90, which make the dimeric molecule chaperone to bind and release substrate proteins like a molecule clamp (reviewed in Pearl and Prodromou [52]). However, dissection of HSP90 function is still restricted by the limited understanding of full-length HSP90 structure and its interaction with cochaperones. 

HSP100 (a.k.a. Clp proteins) is another class of ATP-dependent molecular chaperones. The unique feature of HSP100 family is their capacity to solubilize aggregated proteins and involvement in protein degradations [33, 55]. The best-characterized HSP100 proteins are ClpA from *E. coli* and HSP104 from *S. cerevisiae*. Both ClpA and HSP104 assemble as a hexameric ring with a narrow central pore [35, 56]. This conserved structural organization of HSP100 proteins implies similar functional mechanisms in different organisms. HSP100 plays an essential role in plant survival of severe heat stress [57], but it is absent in some other organisms (ex. Drosophila and vertebrates) that rely on HSP70 and other HSPs to prevent aggregation and accommodate refolding under severe heat stress [58].

### 3.4. sHSP

**Figure 3:**
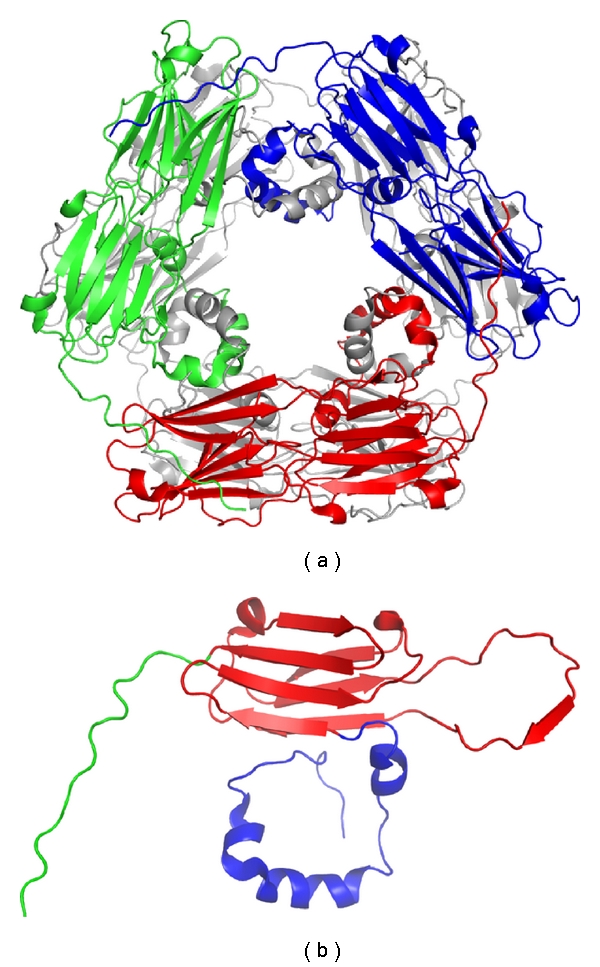
Crystal structure of wheat HSP16.9. (a) The ribbon structure of wheat HSP16.9 reported by Van Montfort et al. (PDB ID: 1GME) [43]. Wheat HSP16.9 is a homo-dodecameric protein consisting of two disk-like layers. The three HSP16.9 dimers in the upper disk are colored in green, blue and red, respectively. The lower disk is colored in gray. (b) The ribbon structure of wheat HSP16.9 monomer. The N-terminal region, *α*-crystallin domain and C-terminal tail are colored in blue, red, and green, respectively.

Small heat shock proteins (sHSPs) are the most ubiquitous HSP subgroup with molecular weights ranging from 12 to 42 kDa. Sequence analysis of sHSPs shows that members of this protein family includes an evolutionarily divergent N-terminal part, followed by a conserved *α*-crystallin domain and a short C-terminal tail (Figure 3) [59]. The number of sHSP genes increases along the evolutionary scale [60]. Single-celled organisms such as bacteria have only one or two sHSP, whereas multicellular organisms have many sHSP in the genome. Particularly, ten separate families of sHSPs have been recognized to be conserved in both monocot and dicot plants, indicating the potential for diversity in sHSP mechanisms [61, 62]. sHSPs encoded by four of these families localize to the cytoplasm, and those encoded by the other six families localize to cellular organelles including nucleus, chloroplasts, mitochondria, endoplasmic reticulum, and peroxisomes [63].

The current model for sHSP chaperone activity was defined based on studies of a cytosolic sHSP family named as Class I sHSPs (sHSP-CI), which represent the most abundant sHSP in plants [64]. The model suggests that sHSP assembles into a large homo-oligomer, which binds denatured proteins in an ATP-independent manner, keeping them in a folding-competent state. Then, it cooperates with ATP-dependent molecular chaperones, such as HSP70 and HSP90, to refold those proteins. Notably, sHSP has a much larger binding stoichiometry than other molecular chaperones, which has led to the speculation that sHSP functions as a reservoir to stabilize the flood of denatured proteins in response to stress [65, 66]. It has been proposed that heat-induced oligomer dissociation is a major mechanism by which plant sHSPs can expose normally inaccessible, hydrophobic client-binding surfaces [60]. Nevertheless, the details about the interactions between sHSP and nonnative proteins and how these nonnative proteins are subsequently refolded are still lacking. This is partially due to limited knowledge on the molecular structure of sHSPs [60]. Among the few solved crystallographic structures of sHSPs is a wheat TaHsp16.9-CI (wHSP16.9, PDB Id: 1GME) [43]. The basic building block of wHSP16.9 is a dimer, which further assembles as a 12-mer consisting of two trimers of dimers (Figure 3). In solution, wHSP16.9 can dissociate into smaller oligomeric states in a temperature dependent manner [43]. On the basis of this observation, it is likely that heat-induced dissociation of sHSP oligomers may expose the hydrophobic patches buried in the oligomeric interface, resulting in binding and stabilization of denatured proteins [43, 60].

## 4. Identification and Characterization of HSPs Associated with Heat Tolerance in Grasses

The presence and role of HSPs in heat tolerance has been examined in various annual grasses cultivated as cereal crops, most of which belong to the genera of rice (*Oryza* sp.), wheat (*Triticum* sp.), maize (*Zea* sp.), sorghum (*Sorghum* sp.), rye (*Secale* sp.), barley (*Hordeum* sp.), and oat (*Avena* sp.). The involvement of HSPs in thermal tolerance has been studied in only a few perennial species such as creeping bentgrass (*Agrostis stolonifera*), fescues (*Festuca* sp.), and orchard grass (*Dactylis glomerata*).  Table 1 summarizes the HSPs reported in the grass family and their tissue specificity, which may play a crucial role in defending each type of tissues against heat stress [85]. How these HSPs are regulated in the defensive and adaptive mechanisms of cereal crops and forage or turf grasses under high temperature are reviewed below.

### 4.1. HSPs Identified in Annual Species Cultivated as Cereal Crops

Expression of HSPs in cereal species was first revealed in some early works in 1980s. In a study [70] examining HSP metabolism in seedlings of five cereal species (common, drurm wheat, barley, rye, and triticale) responding to heat shock at 40°C, inductions of 13 HSPs (14-15, 35–69, 83–99 kDa) were detected. It was also reported that distinct levels of acquired thermal tolerance between wheat varieties were associated with significant quantitative differences in the synthesis of multiple HSPs (16, 17, 22, 26, 33, and 42 kDa) [30].

More thorough characterization of heat-responsive proteins including HSPs benefits from successful application of proteomic-based techniques, particularly two-dimensional gel electrophoresis coupled with mass spectrometry. Lee et al. [74] identified 18 HSPs in a study investigating rice leaf proteome in response to heat stress, including seven HSP70s, three HSP100s, one HSP60, and seven newly induced or highly upregulated sHSPs. Majoul et al. [76] detected upregulation of five sHSPs in a study analyzing the effect of heat stress on hexaploid wheat grain proteome. Using a novel hybrid mass spectrometer (an electrospray ionization-quadrupole linear ion trap (Q-TRAP) combined with nano-HPLC), Süle et al. [72] were able to distinguish six isoforms of a 16.9 kDa sHSP in a proteomic study of barley heat response.

Since each HSP family generally shares high-sequence similarity across diverse cereal species, the anti-HSP antibodies could exhibit relatively broad cross-species activities. Pareek et al. [86] purified and raised highly specific polyclonal antisera against two rice HSPs (104 and 90 kDa), both of which accumulate in response to heat stress. Using these reagents, they detected heat-induced accumulation of the immunological homologues of both HSPs in seedlings of wheat, sorghum, and maize in Western blotting experiments.

### 4.2. HSPs Identified in Perennial Species Cultivated as Forage or Turf Grasses

Park et al. [77] first detected HSPs (97, 83, 70, 40, 25, and 18 kDa) in heat-tolerant and nontolerant variants of creeping bentgrass, a major cool-season turf species. They also found the heat-tolerant variants synthesized two to three additional sHSP (25 kDa). Zhang et al. [78] cloned four classes of HSPs (HSP100, HSP90, HSP70, and sHSPs) that are differentially expressed under heat stress between the two genotypes of fescues, which are widely used as both forage and turf grasses. Cha et al. [80] characterized an endoplasmic reticulum-resident HSP90 gene from orchard grass, whose expression increased during heat stress. This protein functions as a molecular chaperone by preventing thermal aggregation of malate dehydrogenase and citrate synthase. The following section will summarize some of our most recent research on HSP identification in association with heat tolerance in cool-season perennial grass species.

Heat acclimation has been found to induce HSPs in various plant species [28]. In a study [81] examining the effects of heat acclimation (gradual temperature increase) and sudden heat stress (direct temperature increase) on protein synthesis and degradation in a heat-sensitive creeping bentgrass cultivar “Penncross”, it was found that both heat treatments led to the accumulation of several HSPs (23, 36, and 66 kDa); in addition, heat acclimation induced a few extra cytoplasmic HSPs (57 and 54 kDa), which were not present in the unacclimated plants under heat stress. These results suggest that upregulation of HSPs, primarily sHSP, HSP60 or HSP70 based on their molecular weights, is a typical response of perennial grasses to heat stress. Especially, due to the fact that heat acclimation improved heat tolerance of the plants as manifested by lower electrolyte leakage in the leaves of heat-acclimated plants, induction of the two HSP60 proteins during heat acclimation could be related to enhanced thermotolerance in perennial grasses. 

It is known that there exists a positive correlation between cytokinin content and heat tolerance in creeping bentgrass [87], and exogenous application of cytokinins improves heat tolerance [88, 89]. Veerasamy et al. [82] further investigated the effects of exogenous applied zeatin riboside (ZR), a synthetic cytokinin, on protein metabolism associated with heat tolerance in “Penncross”. Improved heat tolerance of ZR-treated plants were manifested by less heat-induced degradation of ribulose-1,5-bisphosphate carboxylase proteins and lower protease activity than untreated plants. Particularly, the expression levels of a few HSPs (32 and 57 kDa) were upregulated in ZR-treated plants under heat stress. These results suggest that some sHSP and HSP60 proteins are among the primary targets in cytokinin regulation of heat tolerance in cool-season perennial grass species.

In order to better understand the roles of HSPs in heat tolerance, a unique C_3_ perennial grass species, rough bentgrass (*Agrostis scabra*) identified in Yellowstone National Park, has been investigated. The thermal *A. scabra* grows actively in the chronically hot soils [90], which may have adopted both heat avoidance and tolerance strategies [91]. The physiological traits associated with superior thermotolerance of the species were described in a few recent publications from our lab. This species could maintain the canopy photosynthesis and respiration rates responding to short-term soil temperature elevation [92]. Its roots tolerate high soil temperature by holding high proportion of alternative respiration [93], low maintenance and ion uptake costs [94], as well as efficient expenditure and adjustment of carbon and nitrogen allocation patterns between growth and respiration [95].

**Figure 4:**
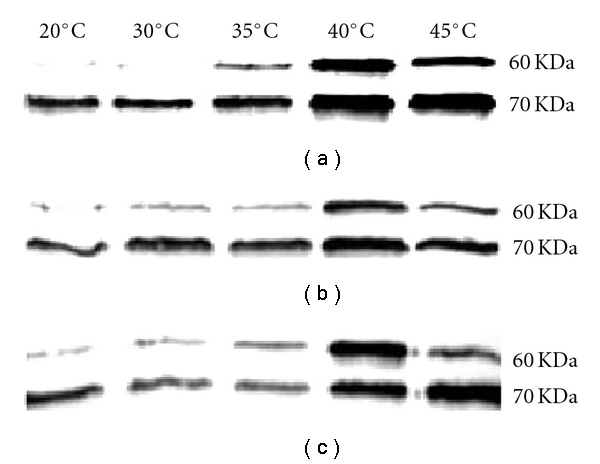
Immunoblots of HSP60 and HSP70 of *Agrostis scabra* (a), creeping bentgrass (*A. stolonifera*) cv. Penncross (b) and cv. L-93 (c) after 3 d of five different temperatures. Equal amounts of protein (18 *μ*g) were loaded in each lane (from [44]).

Heat-induced changes in one-dimensional protein profiles of thermal *A. scabra* were compared to those of* A. stolonifera*. In the shoots, significant protein degradation was observed at 30°C–45°C in “Penncross” and a new heat-tolerant cultivar of creeping bentgrass “L93”, but not until 40°C –45°C in *A. scabra*. Meanwhile, expression of HSPs (23, 32, 36, and 66 kDa) was induced or enhanced at 35°C –45°C in “L-93” and *A. scabra,* but only at 40°C –45°C in “Penncross”. Moreover, stronger expression of HSP60 and HSP70 proteins in the shoots of *A. scabra* or “L-93” than “Penncross” at 35–45°C of 3 d was revealed by immunoblotting (Figure 4). In the roots, heat-induced degradation of proteins including HSPs was mitigated in the thermal species, especially at the extreme temperature (45°C). Immunoblotting detected induction of HSP60 and multiple sHSP (Class I) proteins at elevated temperatures in both species, but the induction in *A. stolonifera* was triggered later under heat stress and/or by higher temperature compared to the thermal species; HSP70 was constitutively produced during heat-shock treatment (2 and 4 h) but prolonged heat stress increased its expression level (24 and 28 h) (Huang, unpublished data). The results from both shoots and roots indicate a correlation between early induction of major HSPs as well as maintenance of them under elevated temperature and better heat tolerance of cool-season perennial grasses. 

A more complete identification and comparison of heat-responsive proteins in the two *Agrostis *grass species contrasting in heat tolerance were achieved through proteomic analysis. Among the hundreds of proteins identified in the leaves is an HSC70, the abundance of which decreased under heat stress in both species [83]. However, the degradation ceased at 2 d in *A. scabra* but continued to 10 d in *A. stolonifera*. It suggests that maintaining production of constitutively expressed HSPs such as HSC70 is important for sustaining grass plant growth under heat stress. In the roots, proteomic analysis revealed the increase of an HSP Sti (stress-inducible protein) in both species under heat stress, which contains two HSP binding motif, three tetratricopeptide repeat and two Sti1 domains [96]. Sti proteins are involved in HSP90 signaling and interaction [97]. As heat-induced accumulation of this protein was earlier and greater in the thermal species compared to heat-sensitive *A. stolonifera*, it indicated that upregulation of HSP90-related proteins such as Sti may contribute to whole-plant thermotolerance in perennial grasses.

The involvement of HSPs in heat tolerance was also determined at the gene level. A suppression subtractive hybridization (SSH) library was constructed by Tian et al. [84] to identify heat-responsive genes for thermal *A. scabra*. In this study, genes of an *HSP20*-like chaperone and an *HSP70* were isolated. Expression of the *HSP70* gene was constitutively expressed under optimum temperature but strongly upregulated under heat stress in both shoots and roots. The HSP20-like chaperone is highly homologous to an HSP20-like chaperone from clover (*Medicago truncatula*) that contains the p23 domain. As p23 is one of the cochaperones of HSP90 and stabilizes the HSP90 heterocomplex [98, 99], enhanced expression of this chaperone gene under heat stress also indicated that upregulation of HSP90-related proteins are important for heat tolerance in perennial grasses, as discussed above in the proteomic study. In another study, using the sequence of the* HSP70 *gene isolated by SSH in *A. scabra* and the reported sequence of a sHSP (*HSP16*) gene in *A. stolonifera*, the expression levels of the two genes were compared between heat-sensitive *A. stolonifera* and thermal *A. scabra* (Huang, unpublished data). The expression of *HSP16* was highly induced in both species at 45°C after 24 h, but the induction was more substantial in the thermal species, whereas, *HSP70* gene was constitutively expressed at optimum temperature but the expression was slightly upregulated at elevated temperatures in both species. The response of *HSP* gene expression to increasing temperature is in accordance with the response of HSP protein abundance to elevated temperature, confirming the direct association of HSPs with heat tolerance in perennial grasses.

Overexpression of genes controlling cytokinins (CKs) synthesis can also modify CKs production in the plants in addition to application of products containing CKs. Transgenic *A. stolonifera* (cv. Penncross) with elevated endogenous CKs level has been successfully generated in our lab and used to study the involvement of CKs in grass tolerance to abiotic stresses including heat stress [79, 100], shade [79], drought [101] and nutrient deficiency [102]. In these plants, the agrobacterium *ipt* gene encoding adenine isopentenyltransferase that catalyzes the key step in *de novo* CK biosynthesis was ligated to either a senescence-activated promoter* SAG12* [103] or a heat-shock promoter *HSP18 *[104]. Delayed leaf senescence under heat stress was observed in both *SAG12-ipt* and *HSP18-ipt* lines [79]. 

**Figure 5:**
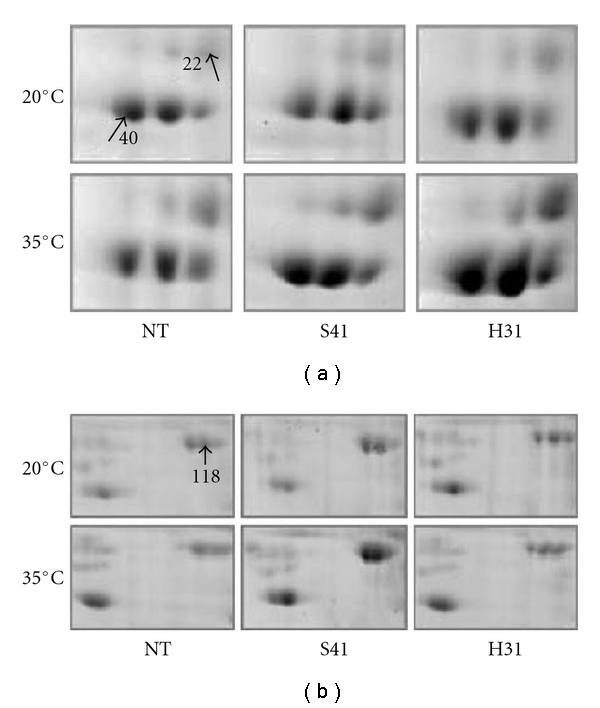
Differential expression of a plastid Hsp90 (no. 22) and a chloroplast Hsp70 (no. 40) proteins in the shoots (a) and an endoplasmic Hsp90 homologue (no. 118) protein in the roots (b) of the nontransgenic plants (NT), *SAG12-ipt* line (S41), and *HSP18-ipt* line (H31) at 10 d of treatment at normal temperature (20°C) or heat stress (35°C) (from [45]).

A *SAG12-ipt* line (S41) and an *HSP18-ipt* line (H31) was selected for a proteomic study to compare genome-wide protein changes associated with differential heat tolerance among *SAG12-ipt*,* HSP18-ipt*, and the nontransformant (NT) lines. A plastid HSP90, a cytoplasmic HSP90, and a chloroplast HSP70 as well as a Rubisco large subunit-binding protein subunit *α* (chHSP60) were identified in the shoots, and two endoplasm HSP90 homologues were identified in the roots (Figure 5). Specifically, in the shoots, the abundance of the plastid HSP90 increased 2.8-fold only in S41 whereas the abundance of the cytoplasmic HSP90 decreased 70% only in NT under heat stress; the chloroplast HSP70 was upregulated 1.5-fold and 2.0-fold in S41 and sH31, respectively, but not in NT (Figure 5(a)). An increase in the abundance of the Rubisco large subunit-binding protein subunit *α* was detected only in NT but not in either *ipt*-transgenic line. Upregulation of this chHSP60 protein indicates that proper folding of Rubisco proteins may be disturbed by heat stress thus more chHSP60s are required as their primary function is to facilitate Rubisco folding. In the roots, increased abundance of both endoplasmic HSP90 homologues was only observed in S41 under heat stress (Figure 5(b)). The results confirmed the regulatory role of CK in HSP metabolism for heat tolerance by inhibition of its degradation or stimulation of its production. HSPs belonging to the same group but assigned to different subcellular locations can be regulated distinctively, suggesting they may possess distinct functional mechanisms for heat tolerance.

## 5. Incorporation of HSPs for Improving Heat Tolerance

Conventional breeding contributed substantially to the genetic improvement of grass germplasms in the last century [105]. For instance, specific HSPs are involved in breeding heat-tolerant maize [106]. When crossing a heat-tolerant maize line (ZPBL 1304) that synthesizes a 45 kDa HSP and a heat-sensitive line (ZPL 389) that does not synthesize this protein, synthesis of the 45 kDa HSP was observed in F_2_ plants that displayed an increased ability to recover from heat stress [107].

However, in most cases, germplasm screening for heat tolerance relies on field and whole-plant techniques, which are less efficient and sensitive due to environmental interactions [108]. Continuous efforts have been devoted to developing rapid and accurate procedures that allow simultaneous screening of large numbers of genotypes in order to breed heat-tolerant grass for use in hot and humid areas [12]. Recent progress in genetic manipulation of plants opens up opportunities for incorporating cellular and molecular techniques into grass improvement [109]. The technology exists to make pinpoint genetic changes to grass using marker-assisted selection or direct gene transfer by biolistic transformation and agrobacterium-mediated transformation [110–112]. 

A few successful cases on incorporation of* HSP* genes to improve heat tolerance were reported in rice. For example, enhanced thermotolerance was achieved in transgenic rice overexpressing an arabidopsis *HSP101* gene [113]. Overexpression of a rice chloroplast *sHSP *(*Oshsp26*) gene conferred better tolerance not only to heat stress but also to oxidative stress in *E. coli* [114], and overexpression of sHSP17.7 confers both heat tolerance and UV-B resistance to rice plants [115]. The effectiveness of this strategy in breeding heat-tolerant perennial grasses needs to be further validated.

In conclusion, heat tolerance of both annual and perennial grasses encompasses an orderly, dynamic and complex regulatory system of different groups of HSPs. Evidences are available on the association of early induction and persistent maintenance of HSPs under elevated temperature with better heat tolerance in grasses. Manipulating genes controlling HSP production may be beneficial for breeding heat-tolerant grass genotypes.

## Figures and Tables

**Table 1:** Tissue-specific expression of five families of HSPs in cereal and forage and turf grasses responding to heat stress.

*HSP Family*	*Tissue*	*Cereal Species*
HSP100	Leaf	Wheat [67]
	Root	Wheat [67]
	Seed	Maize [68], Rice [68], Wheat [68]

HSP90	Seed	Maize [69]

HSP70	Leaf	Wheat [70]
	Root	Maize [69], Wheat [70]
	Seed	Wheat [71]

HSP60	Leaf	Maize [69]
	Root	Barley [70], Maize [69], Rye [70], Wheat [70]
	Seed	Maize [69]

sHSP	Leaf	Barley [72], Maize [69, 73], Rice [74], Wheat [30, 70]
	Root	Maize [69], Wheat [70]
	Seed	Pearl millet [75], Sorghum [75], Wheat [71, 76]

*HSP Family*	*Tissue*	*Forage and Turf Species*

HSP100	Leaf	Creeping bentgrass [77], Fescue [78]

HSP90	Leaf	Creeping bentgrass [77, 79], Fescue [78], Orchardgrass [80]
	Root	Creeping bentgrass [79]

HSP70	Leaf	Creeping bentgrass [77, 79, 81–84], Fescue [78]
	Root	Creeping bentgrass (unpublished)

HSP60	Leaf	Creeping bentgrass [79, 81, 82]
	Root	Creeping bentgrass (unpublished)

sHSP	Leaf	Creeping bentgrass [77, 81, 82, 84], Fescue [78]
